# Functional Connectivity Dynamics Altered of the Resting Brain in Subjective Cognitive Decline

**DOI:** 10.3389/fnagi.2022.817137

**Published:** 2022-06-24

**Authors:** Yi-Chia Wei, Yi-Chia Kung, Wen-Yi Huang, Chemin Lin, Yao-Liang Chen, Chih-Ken Chen, Yu-Chiau Shyu, Ching-Po Lin

**Affiliations:** ^1^Institute of Neuroscience, National Yang Ming Chiao Tung University, Taipei, Taiwan; ^2^Community Medicine Research Center, Chang Gung Memorial Hospital, Keelung, Taiwan; ^3^Department of Neurology, Chang Gung Memorial Hospital, Keelung, Taiwan; ^4^College of Medicine, Chang Gung University, Taoyuan, Taiwan; ^5^Department of Psychiatry, Chang Gung Memorial Hospital, Keelung, Taiwan; ^6^Department of Medical Imaging and Radiological Sciences, Chang Gung University, Taoyuan, Taiwan; ^7^Department of Radiology, Chang Gung Memorial Hospital, Keelung, Taiwan; ^8^Department of Nursing, Chang Gung University of Science and Technology, Taoyuan, Taiwan

**Keywords:** subjective cognitive decline (SCD), preclinical dementia, resting state functional MRI, dynamic functional connectivity, mdALFF, dynamics

## Abstract

**Background:**

Subjective cognitive decline (SCD) appears in the preclinical stage of the Alzheimer's disease continuum. In this stage, dynamic features are more sensitive than static features to reflect early subtle changes in functional brain connectivity. Therefore, we studied local and extended dynamic connectivity of the resting brain of people with SCD to determine their intrinsic brain changes.

**Methods:**

We enrolled cognitively normal older adults from the communities and divided them into SCD and normal control (NC) groups. We used mean dynamic amplitude of low-frequency fluctuation (mdALFF) to evaluate region of interest (ROI)-wise local dynamic connectivity of resting-state functional MRI. The dynamic functional connectivity (dFC) between ROIs was tested by whole-brain-based statistics.

**Results:**

When comparing SCD (*N* = 40) with NC (*N* = 45), mdALFF_mean_ decreased at right inferior parietal lobule (IPL) of the frontoparietal network (FPN). Still, it increased at the right middle temporal gyrus (MTG) of the ventral attention network (VAN) and right calcarine of the visual network (VIS). Also, the mdALFF_var_ (variance) increased at the left superior temporal gyrus of AUD, right MTG of VAN, right globus pallidum of the cingulo-opercular network (CON), and right lingual gyrus of VIS. Furthermore, mdALFF_mean_ at right IPL of FPN are correlated negatively with subjective complaints and positively with objective cognitive performance. In the dFC seeded from the ROIs with local mdALFF group differences, SCD showed a generally lower dFC_mean_ and higher dFC_var_ (variance) to other regions of the brain. These weakened and unstable functional connectivity appeared among FPN, CON, the default mode network, and the salience network, the large-scale networks of the triple network model for organizing neural resource allocations.

**Conclusion:**

The local dynamic connectivity of SCD decreased in brain regions of cognitive executive control. Meanwhile, compensatory visual efforts and bottom-up attention rose. Mixed decrease and compensatory increase of dynamics of intrinsic brain activity suggest the transitional nature of SCD. The FPN local dynamics balance subjective and objective cognition and maintain cognitive preservation in preclinical dementia. Aberrant triple network model features the dFC alternations of SCD. Finally, the right lateralization phenomenon emerged early in the dementia continuum and affected local dynamic connectivity.

## Introduction

Subjective cognitive decline (SCD) refers to individuals' perceived decline in memory or other cognitive abilities relative to their previous level of performance in the absence of objective neuropsychological deficits (Jessen et al., 2014). SCD is considered a deviation from normal aging and representative for the late preclinical stage of Alzheimer's disease (AD) (Sperling et al., 2011). Cognitive changes in SCD are characterized by subtle cognitive decline and compensatory cognitive efforts (Jessen et al., 2014).

Magnetic resonance imaging (MRI) is an essential noninvasive tool in cognitive neuroscience. Functional MRI (fMRI) detects blood oxygenation level-dependent (BOLD) signals to reflect neuronal activities in the human brain (Grady, 2012). In addition, resting-state fMRI (rs-fMRI) revealed the intrinsic brain activity of human brain, networks of functional connections, and their relationships with neuropsychiatric diseases (Zhang and Raichle, 2010).

### Early Local, Late Global Connectivity Changes in AD Spectrum

In neurodegeneration of the AD continuum, pathological tau deposition is closely related to cognitive performance (Hanseeuw et al., 2019) and is a good tracer of disease progression (Brier et al., 2016). The topological similarity between functional disconnection and tau deposition reflects the pathological functional coupling in AD (Ossenkoppele et al., 2019; Franzmeier et al., 2020). Measuring changes in functional connectivity is a noninvasive approach for understanding pathogenesis of AD neurodegeneration. Along the trajectory of disease progression from preclinical stage to AD, functional connectivity decreases earlier before structural destruction (Jack et al., 2010; Sperling et al., 2011). Therefore, functional disconnection can be measured in SCD, but not yet the gray matter volume reduction (Sun et al., 2016; Dong et al., 2018; Parker et al., 2020). In addition, the functional disconnection develops locally in the early stage (Sun et al., 2016) before the late global disconnection (Liu et al., 2014). For example, in a hierarchical comparison of normal people, SCD, mild cognitive impairment (MCI), and patients with AD, only local functional disconnection developed in SCD, whereas mixed local and global disconnection started in MCI and shifted to fully global compensation in AD (Wang et al., 2019). Therefore, studying local functional connectivity reveals the earliest changes in the brain of preclinical dementia.

### Local Functional Activation in SCD

Local functional activation was commonly assessed by recording the spontaneous activity at the resting brain. At first, fluctuations of BOLD signals at 0.01–0.1 Hz were used to describe network features of the resting-state default mode of the brain (Fransson, 2005) and its responses to cognitive tasks (Fransson, 2006). More recently, the combined study of 18F-fluorodeoxyglucose positron emission tomography (FDG-PET) and rs-fMRI found the coupling of glucose metabolism and the amplitude of low-frequency fluctuation (ALFF) of BOLD signals. The two methods are consistent for recording brain activity (Jiao et al., 2019).

Previously, ALFF has been applied to characterize disease-related regional functional changes in the brain, such as those in the elderly with AD (He et al., 2007) and the children with attention deficit hyperactivity disorder (ADHD) (Zang et al., 2007). In SCD, when compared to healthy controls, the distinct local connectivity patterns are increased ALFF values in bilateral inferior parietal lobule (IPL), right inferior occipital gyrus, right middle occipital gyrus, right superior temporal gyrus (STG), and right cerebellar posterior lobe (Sun et al., 2016), as well as decreased ALFF values in the precuneus, anterior cingulate cortex, and cerebellum (Yang et al., 2018). In addition, the patterns of regional ALFF and fractional ALFF (fALFF) distinguished SCD, MCI, and AD from normal people in the machine learning model (accuracy 76–92%) (Yang et al., 2018). Therefore, measuring ALFF is a feasible way to characterize the early brain changes in preclinical dementia.

### Why Studying Dynamic Connectivity in SCD

Currently, studies of static functional connectivity (sFC) (Viviano and Damoiseaux, 2020; Wang et al., 2020) are much abundant than that of dynamic functional connectivity (dFC) in SCD (Xie et al., 2019; Dong et al., 2020; Yang et al., 2020; Chen et al., 2021). However, brain activity is context-sensitive and activity-dependent. The intrinsic brain activity is dynamic and fluctuates overtimes. sFC may not show the full picture of the changes in the brain integrated states. In contrast, dFC can be more comprehensive than sFC in characterizing functional features of neurodegeneration. For example, dFC outperformed sFC in classifying AD from controls [area under the receiver operating characteristic curve (AUROC) 0.82 for sFC matrices and 0.84 for dFC] (de Vos et al., 2018).

In addition, dFC provides complementary information to sFC, such as in aging-related functional connectivity changes. The dFC studies of rs-fMRI showed posterior-attenuated and anterior-enhanced local hub dynamics in aging people (Zhang et al., 2017), echoing the posterior–anterior shift in aging (PASA) model that describes asymmetric changes in aging brains by posteriorly-decreased and anteriorly-increased BOLD signals in tasks fMRI (Davis et al., 2008). However, the directions of the linear gradients were opposite for sFC and dFC, suggesting that sFC and dFC provided complementary information of intrinsic brain activity alterations in the aging brains (Zhang et al., 2017).

Because the cognitive decline of SCD is subtle and studying the dynamics of functional connectivity reveals more details of the early neurodegeneration, we compared the local dynamic connectivity in rs-fMRI between SCD and normal healthy control by the mean dynamic amplitude of low-frequency fluctuation (mdALFF). We also looked for their outward dFC to seek emerging alterations of the intrinsic brain function of preclinical dementia.

## Methods

### Definition of Grouping

Subjective cognitive decline was defined by the two criteria of the Subjective Cognitive Decline Initiative Working Group: (1) self-experience of a persistent decline of cognitive capacity when compared to a previously normal cognitive status, and the change did not correlate with any acute event, and (2) the performance of standardized cognitive tests met the expected levels (Molinuevo et al., 2017). In our study setting, grouping to SCD required: (1) having SCCs, defined by a self-reported AD8 score ≥2 points (Galvin et al., 2005; Yang et al., 2011; Wei et al., 2019), and (2) normal cognition, defined by a MoCA score higher than the score of age- and education-matched means minus one standard deviation (Rossetti et al., 2011). In contrast, the normal control (NC) group included the cognitively normal participants without SCCs by an AD8 score of 0–2.

### Measurements

AD8 is a brief measure by 8 questions to detect cognitive impairment regarding the daily cognitive abilities of judgment, interest, repeats, appliances, orientation, finance management, appointment remembering, and the consistency of cognitive changes. Endorsement of a change to each cognitive problem in the last several years scores 1 point. A total score equal to or over 2 points represents having subjective cognitive complaints (SCCs) (Galvin et al., 2005, 2006). The original English version of the AD8 questionnaire was created by Galvin et al. in 2005 (Galvin et al., 2005) and can be applied in informant-based (Galvin et al., 2006) or self-report manners (Galvin et al., 2007). We used the Traditional Chinese version of the AD8 questionnaire, which was well validated (Yang et al., 2011). Because self-endorsed decline usually occurs earlier than informant confirmed decline (Caselli et al., 2014), we applied AD8 in a self-report manner for detecting SCCs (Wei et al., 2019, 2021).

The Montreal Cognitive Assessment (MoCA) score determined their objective cognitive performance. Impairment of cognitive performance was defined by a MoCA score lower than one standard deviation below the mean of age- and education-adjusted norms (Rossetti et al., 2011). The other cognitive tests in this study included the Mini-Mental State Examination (MMSE) (Shyu and Yip, 2001), digit symbol coding (DSC), digit span test (DST), letter-number sequencing (LNS), category fluency (CF), and facial memory test (FMT). We also use the Hospital Anxiety and Depression Scale (HADS), which contained anxiety subscale (HADS-A) and depression subscales (HADS-D), for evaluating anxiety and depression tendency (Bjelland et al., 2002).

### Participant Enrollment

This work was a part of the Northeastern Taiwan Community Medicine Research Cohort (NTCMRC; identifier on ClinicalTrials.gov: NCT04839796) conducted by the Community Medicine Research Center of the Chang Gung Memorial Hospital in Keelung, Taiwan. The cohort was launched in Northeastern Taiwan in 2012 and started cognitive and brain imaging recording in 2018. The Institutional Review Board of Chang Gung Memorial Hospital approved this study (approval no. 201600580B0, 201600270B0, 201600269B0, 201901350B0, 201901353B0, 201901352B0, and 200600269B0). All the participants signed informed consent before entering this study.

During 2018–2020, we enrolled healthy, right-handed, older adults aged over 50 years. Participants with major organ failure, including heart failure, renal failure, moderate-to-severe liver disease, and active thyroid diseases, were not recruited. In the initial screening, we excluded the participants who fulfilled the criteria of MCI (Petersen et al., 1999; Winblad et al., 2004) or dementia (Mckhann et al., 2011) or had other brain disorders, including stroke, epilepsy, brain tumor, traumatic brain injury, and developmental neurological diseases. Through the Mini-International Neuropsychiatric Interview (Lecrubier et al., 1997), we further excluded those participants with psychiatric disorders. Brain structural MRI screened the structural lesion(s) and excluded those participants before going through the rs-fMRI analysis.

**Figure 1:**
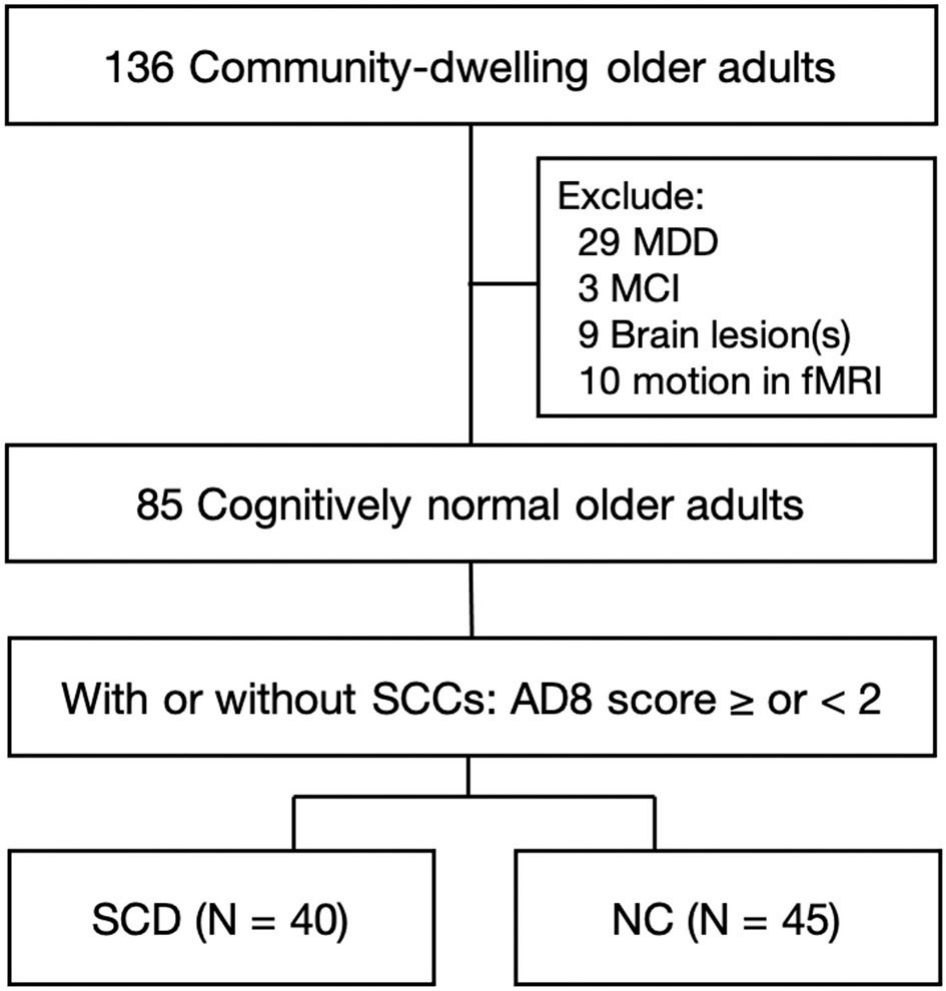
Flow chart of enrollment. Healthy older adults aged over 50 years were enrolled from the communities. Initial screening excluded the participants who had a current state or previous history of MDD, met the criteria of MCI, had brain lesions in structural MRI, or had head motion during fMRI scans. These 85 cognitively normal older adults were further divided into the group of SCD and NC, based on having or not having SCCs by an AD8 score ≥2 or <2 points. MDD, major depressive disorder; MCI, mild cognitive impairment; SCCs, subjective cognitive complaints; SCD, subjective cognitive decline; NC, normal control.

From 136 community-dwelling healthy older adults, we excluded 29 participants for current or history of major depressive disorder, 3 participants who met the criteria of MCI, 9 participants for brain lesions in the structural MRI, and the other 10 for motion found in fMRI image preprocessing. Then, having or not of SCCs divided these 85 cognitively normal healthy older adults into SCD group (*N* = 40) and NC group (*N* = 45) (Figure 1).

The 85 enrolled participants had a mean age of 65.47 ± 5.69 years, a female-to-male ratio of 1.36 (49 women and 36 men), and 10.10 ± 4.19 years of school education. Between-group comparisons confirmed the equality of age, sex, and education level between SCD and NC (Table 1). The SCD group had higher degree of SCCs (AD8 score 4.03 ± 1.82 vs. 0.18 ± 0.39, *p* < 0.001) and poorer cognitive performance in MoCA (23.85 ± 3.29 vs. 26.31 ± 3.52, *p* = 0.001), LNS (*p* = 0.021), CF of animal (*p* = 0.004), and color (*p* = 0.028) than the NC group. Compared to NC, SCD also had higher tendency of anxiety (HADS-A score 5.40 ± 3.21 vs. 2.84 ± 2.84, *p* < 0.001) and depression (HADS-D score 5.45 ± 3.36 vs. 2.80 ± 3.29, *p* < 0.001) (Table 1).

**Table 1:** Between group comparison.

	**SCD (*N =* 40)**	**NC (*N =* 45)**	***p*-value**
**Basic information**			
Age	64.93 ± 5.65	65.96 ± 5.75	0.408
Sex, female	27 (67.5%)	22 (48.9%)	0.085
Education, year	9.80 ± 4.52	10.38 ± 3.90	0.529
**Cognition**			
AD8 (0-8)	4.03 ± 1.82	0.18 ± 0.39	<0.001^*^
MMSE (30-0)	28.13 ± 1.45	28.38 ± 1.81	0.484
MoCA (30-0)	23.85 ± 3.29	26.31 ± 3.52	0.001^*^
DSC	53.18 ± 17.31	60.31 ± 22.83	0.115
DST-forward	12.18 ± 2.34	12.42 ± 2.24	0.629
DST-backward	6.21 ± 3.12	7.36 ± 2.64	0.071
LNS	8.26 ± 2.55	9.76 ± 3.19	0.021^*^
CF-animal	16.05 ± 4.81	19.18 ± 4.86	0.004^*^
CF-fruit	13.26 ± 3.39	14.11 ± 3.02	0.225
CF-color	12.21 ± 4.09	14.33 ± 4.58	0.028^*^
CF-city	18.23 ± 5.81	20.02 ± 5.64	0.156
FMT	34.03 ± 4.06	35.89 ± 4.80	0.060
**Anxiety and depression**			
HADS-A (0–21)	5.40 ± 3.21	2.84 ± 2.84	<0.001^*^
HADS-D (0–21)	5.45 ± 3.36	2.80 ± 3.29	<0.001^*^

* *Statistical significance at p < 0.05. Data were given as mean ± standard deviation or n (%). Ranges of the rating scales were annotated from normal to abnormal. Clinical meanings of the rating scale were AD8 for subjective cognitive complaints, MMSE and MoCA for general cognitive assessment, HADS-A for anxiety, and HADS-D for depression*.

*NC, normal control; SCD, subjective cognitive decline; MMSE, Mini-Mental State Examination; MoCA, Montreal Cognitive Assessment; DSC, digit symbol coding; DST, digit span test; LNS, letter-number sequencing; CF, category fluency; FMT, facial memory test; HADS-A, Hospital Anxiety and Depression Scale—Anxiety subscale; HADS-D, Hospital Anxiety and Depression Scale—Depression subscale*.

### Image Data Acquisition

Magnetic resonance imaging data were collected using a 3T Siemens Skyra system (Erlangen, Germany) and a 20-channel head-neck coil at Keelung Chang Gung Memorial Hospital. High-resolution T1-weighted anatomical images (3D-MPRAGE with 256 × 256 × 256 matrix size, 1 mm^3^ isotropic cube, flip angle (FA) = 8, repeat time (TR) = 2200 ms, echo time (TE) = 2.45 ms, and inverse time = 900 ms) were acquired before the functional scans for localization reference. Customized cushions were used to minimize head motion during each scan. rs-fMRI scans were subsequently acquired using a single-shot, gradient-recalled echo-planar imaging sequence (TR/TE/FA = 2,500 ms/27 ms/90, field of view = 220 mm, matrix size = 64 × 64, 35 slices with 3 mm thickness, 200 measurements) aligned along the anterior commissure-posterior commissure line, thus allowing whole-brain coverage.

### Image Preprocessing

All the fMRI data were preprocessed by the analysis of functional neuroImages (AFNI) (Cox, 1996), FMRIB Software Library (FSL) (Smith et al., 2004), and statistical parametric mapping (SPM) (Friston, 2007). The data with excessive motion resulting in translation >2 mm, rotation >2°, and a mean frame displacement exceeding 0.5 mm were excluded. The first 10 volumes were first deleted to ensure that the data were acquired with a steady-state signal. In the preprocessing stage, all the fMRI datasets were subjected to motion correction with the Friston 24-parameter model (Friston et al., 1996), skull-stripping, slice-timing, despiking, and detrending. For the anatomical information, native fMRI images were registered to the native T1-weighted image and segmented into white matter, gray matter, and cerebrospinal fluid. The fMRI datasets were spatially normalized to a standard Montreal Neurological Institute (MNI) template and resampled to an isotropic resolution of 2 × 2 × 2 mm^3^. Then, the linear detrending was applied to eliminate any signal drift induced by system instability. Finally, the effects of nuisance regressors, including the six motion parameters, respiration/cardiac pulsations, white matter, and cerebrospinal fluid, were removed from the preprocessed datasets. The preprocessed data were temporally bandpass filtered between 0.01 and 0.1 Hz and then smoothed with a Gaussian kernel (full width at half maximum = 6 mm) to improve the signal-to-noise ratio.

### Dynamic Analysis for Functional Metrics

#### Software and Atlas

The ROI-based dynamic metrics were generated by the DynamicBC toolbox (version 2.2) (Liao et al., 2014), with a sliding-window approach (window size = 50 TRs, step size = 10 TRs) on the rs-fMRI dataset referencing our previous study (Kung et al., 2019). The window length follows the criterion (Leonardi and Van De Ville, 2015) with adequate sampling; furthermore, it is long enough with stable and reliable results and short enough to detect quick changes. The ROI-based analysis was performed in each window, resulting in multiple time-varying dynamic metrics. The functional metrics were calculated on the 300 ROI parcellation (Seitzman et al., 2020), including cortical, subcortical, and cerebellum structures with 14 predefined resting-state network parcellations. We excluded 27 ROIs belonging to the undefined network and used the 273 ROIs for the following analysis. Meanwhile, the anatomic annotation of the ROIs was obtained by referring to the Automated Anatomical Labeling (AAL) atlas (Tzourio-Mazoyer et al., 2002).

#### Local Dynamic Connectivity: mdALFF Analysis

We transformed voxel-wise ALFF into ROI-wise dynamic metrics to study local dynamic connectivity and then applied it to predefined functional networks. In detail, (1) we used the fast Fourier transform (FFT) (parameters: taper percent = 0, FFT length = shortest) of the filtered time series as the power spectrum. Then, the ALFF as the average square root of the power spectrum across 0.01–0.08 Hz was calculated for each voxel (Zang et al., 2007). (2) Next, the ALFF map was divided by the global mean of whole-brain ALFF to mean ALFF (mALFF). Then, the values of all the voxels in each ROI were averaged as ROI-wise mALFF. (3) After generating the ROI-wise mALFF map, we combined the sliding-window technique to summarize the mdALFF metrics of whole-brain ROIs. The mean and variance of mALFF across windows were obtained as mdALFF_mean_ and mdALFF_var_, respectively (Liao et al., 2019). (4) Finally, the mdALFF values of the 273 ROIs were aggregated into the predefined functional network (Seitzman et al., 2020).

#### Extended Dynamic Connectivity: dFC Analysis

Next, we examined the dynamics of inter-regional functional connectivity extended from the regions with significant mdALFF group differences. The ROIs with significant differences of mdALFF_mean_ or mdALFF_var_ between the SCD and NC groups were taken as seeds. The dFC from the seeds was calculated as follows: (1) The BOLD times series of all voxels within each ROI were averaged for calculating the Pearson's correlation coefficients in a pairwise manner of the seed ROI to the whole-brain ROIs. (2) These correlation coefficients, considered the dFC, were transformed into *Z-*scores using Fisher's *Z* formula for statistical analysis. (3) Then, the temporal series of dFC values from each sliding window were averaged and yielded the mean of dFC (dFC_mean_) and the variance of dFC (dFC_var_) for each participant.

### Statistical Analysis

#### Comparing mdALFF Between SCD and NC

The group difference of the mean and variance of the mdALFF values between the SCD and NC groups were examined by a generalized linear model with adjustment for age, sex, education, and frame displacement as covariates and *post hoc* analysis with the Tukey's method. Statistical significance was considered at a confidence level of 99.5% by a *p* < 0.005. Next, we used the false discovery rate (FDR) to test the statistical significance in multiple comparisons (Benjamini and Hochberg, 1995). The FDR correction was applied in two ways: first, whole-brain correction based on the 273 ROIs; second, network-based correction using FDR to check multiple comparisons among the ROIs within each functional network. The reason for using network-based correction was that large-scale functional connectivity organizes internally and works together for specific functions, and each network was considered a processing system (Power et al., 2011).

#### Comparing dFC Between SCD and NC

To compare the difference in dFC between the SCD and NC groups, we used the network-based statistic (NBS) to control the family-wise error rate in identifying group difference for the clusters of connected connections. A *p*-value below 0.05 with 1,000 permutations of original data was considered statistically significant (Zalesky et al., 2010).

#### Correlation Analysis of mdALFF Values and Clinical Indices

A partial correlation analysis adjusted for age, sex, education, and frame displacement examined the relationship between mdALFF and the clinical variables. The mdALFF_mean_ and mdALFF_var_ were tested for correlations to the scores of AD8, MMSE, MoCA, DSC, DST, CF categories, FMT, HADS-A, and HADS-D using the data from all the participants. A correlation coefficient was considered statistically significant by a *p*-value < 0.05 after FDR corrections for multiple comparisons.

In addition, we tested the mdALFF clinical correlation in separated groups of SCD and NC. The partial correlation coefficients of each group underwent Fisher's Z-transformation and then two-tailed t-tests for group differences. FDR further examined the group comparison of correlation, and an FDR-corrected p-value < 0.05 was defined as statistical significance.

## Results

### Local Dynamic Connectivity mdALFF

**Figure 2:**
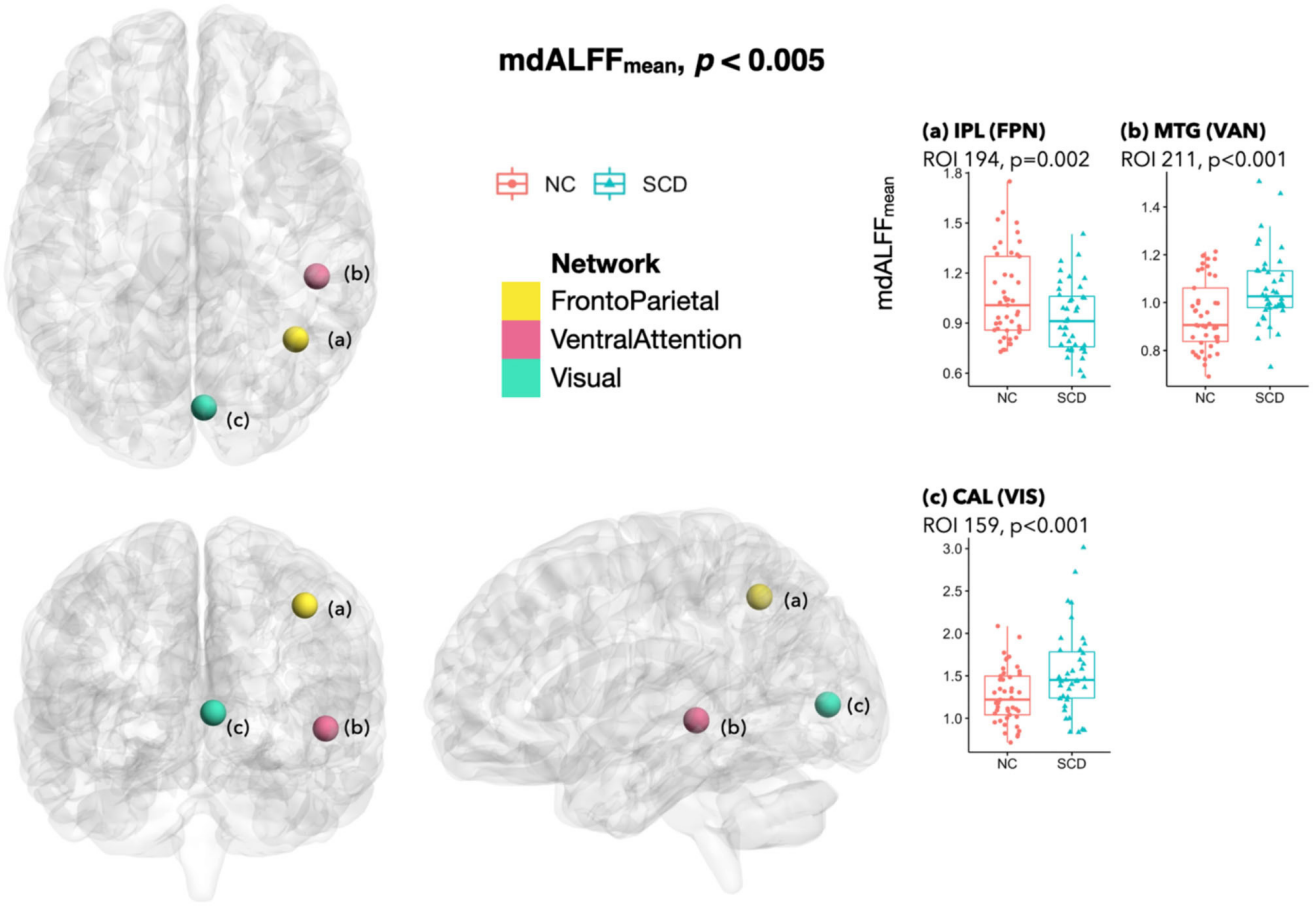
Differences of mdALFF_mean_ between SCD and NC. When comparing the SCD group with the NC group, a significant decrease of mdALFF_mean_ at a *p* < 0.005 was found at ROI(a) in right IPL of FPN. In contrast, the mdALFF_mean_ increased in ROI(b) in right MTG of VAN and ROI(c) in right CAL of VIS. In addition, a marginal decrease of mdALFF_mean_ was observed at a cFPN ROI in right lobule VI, two cDMN ROIs in left Crus I/II, and a AUD ROI in right Rolandic area (*p*-value between 0.005–0.01; not shown in the figure). The anatomical location of each ROI was referred to the Automated Anatomical Labeling atlas (Tzourio-Mazoyer et al., 2002), and their functional network attribution was classified by Seitzman et al. (Seitzman et al., 2020). SCD, subjective cognitive decline; NC, normal control; mdALFF_mean_, mean of the mean dynamic amplitude of low-frequency fluctuation; ROI, region of interest; IPL, inferior parietal lobule; FPN, frontoparietal network; MTG, middle temporal gyrus; VAN, ventral attention network; CAL, calcarine fissure and surrounding primary visual cortex; VIS, visual network; cFPN, frontoparietal network in the cerebellar regions; cDMN, default mode network in the cerebellar regions; AUD, auditory network.

Comparing the local dynamic connectivity between the SCD and NC groups, mdALFF_mean_ decreased at ROI(a) that located in the right IPL and belonged to the frontoparietal network (FPN) (*p* = 0.002). In contrast, we observed an increase of mdALFF_mean_ at ROI(b) in the right middle temporal gyrus (MTG) of the ventral attention network (VAN) (*p* < 0.001) and ROI(c) in the right calcarine fissure and surrounding cortex (CAL) of the visual network (VIS) (*p* < 0.001) (Figure 2).

**Figure 3:**
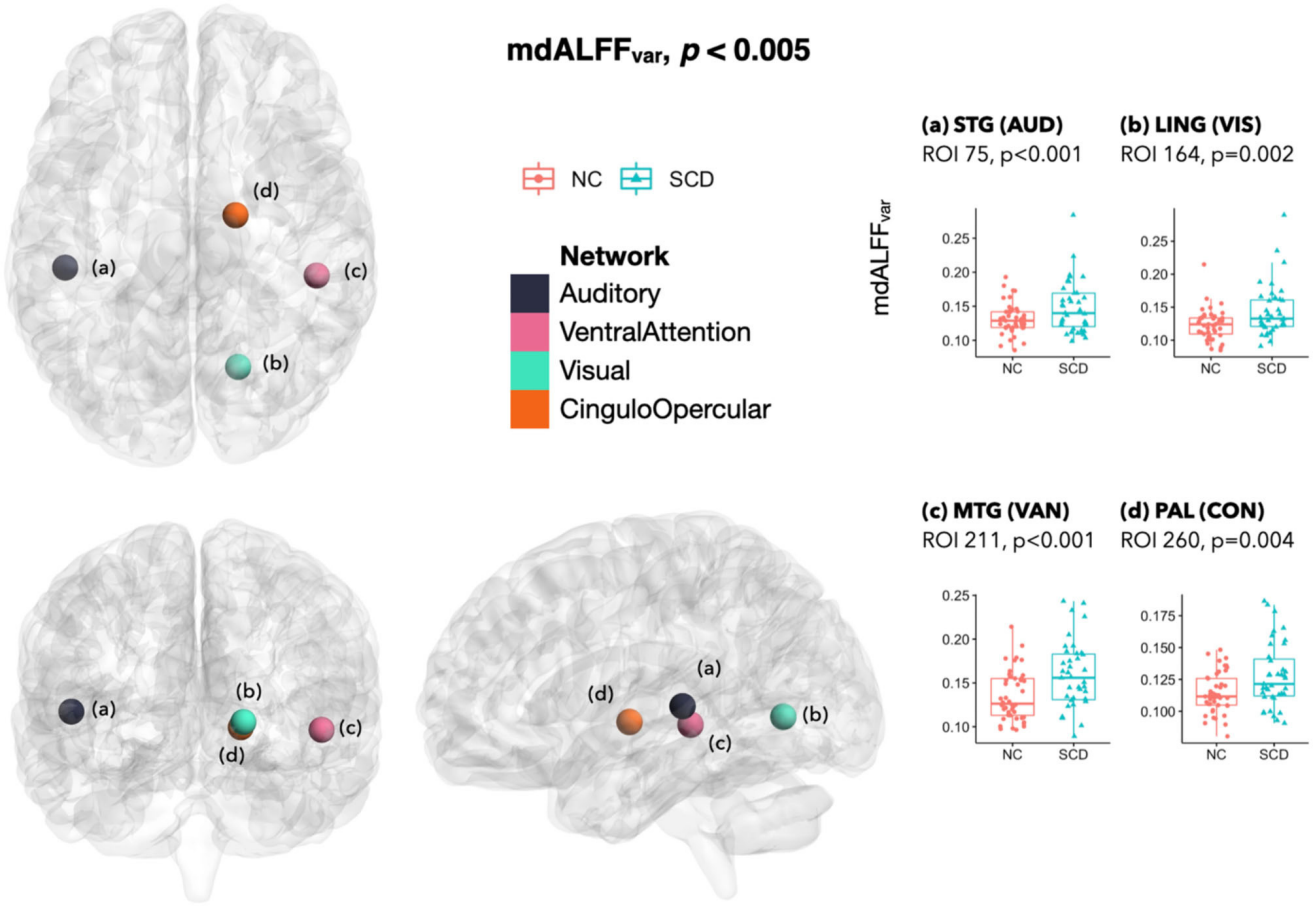
Differences of mdALFF_var_ between SCD and NC. The mdALFF_var_ was higher in SCD than NC at ROI **(A)** in left STG of AUD, ROI **(B)** in right LING of VIS, ROI **(C)** in right MTG of VAN, and ROI **(D)** in right PAL of CON. In the SCD group, marginal changes of mdALFF_var_ included an increase at a VIS ROI in right CAL (*p* = 0.005) and a decrease at a cFPN ROI in left Crus I (*p* = 0.006) (not in the figure). mdALFF_var_, variance of the mean dynamic amplitude of low-frequency fluctuation; SCD, subjective cognitive decline; NC, normal control; ROI, region of interest; STG, superior temporal gyrus; AUD, auditory network; LING, lingual gyrus; VIS, visual network; MTG, middle temporal gyrus; VAN, ventral attention network; PAL, pallidum of lenticular nucleus; CON, cingulo-opercular network; CAL, calcarine fissure and surrounding primary visual cortex; cFPN, frontoparietal network in the cerebellar regions.

In the SCD group, mdALFF_var_ was higher at ROI(a) in left STG of the auditory network (AUD) (*p* < 0.001), ROI(b) in right lingual gyrus (LING) of VIS (*p* = 0.002), ROI(c) in right MTG of VAN (*p* < 0.001), and ROI(d) in right lenticular nucleus pallidum (PAL) of the cingulo-opercular network (CON) (*p* = 0.004) (Figure 3).

In addition to the significant local dynamic connectivity changes, there were some marginal mdALFF group differences. For example, the SCD had marginal mdALFF_mean_ decreases than the NC at an ROI of right Rolandic operculum (ROL) that belonged to AUD (*p* = 0.006), an ROI in right lobule VI that belonged to the cerebellar regions of frontoparietal network (cFPN) (*p* = 0.008), and two ROIs in left Crus I/II of the cerebellar regions of default mode network (cDMN) (*p* = 0.006 and 0.006; not shown in figures). Moreover, mdALFF_var_ marginally increased at a VIS ROI in the right CAL (*p* = 0.005; not shown in figures), but marginally decreased at a cFPN ROI in the left Crus I (*p* = 0.006) in SCD than NC.

The network-based FDR for mdALFF values was significant for mdALFF_mean_ at right MTG of VAN and right CAL of VIS, as well as for mdALFF_var_ at right MTG of VAN and left STG of AUD. The whole-brain ROI-wise FDR was not significant.

### Difference of dFC Between SCD and NC

**Figure 4:**
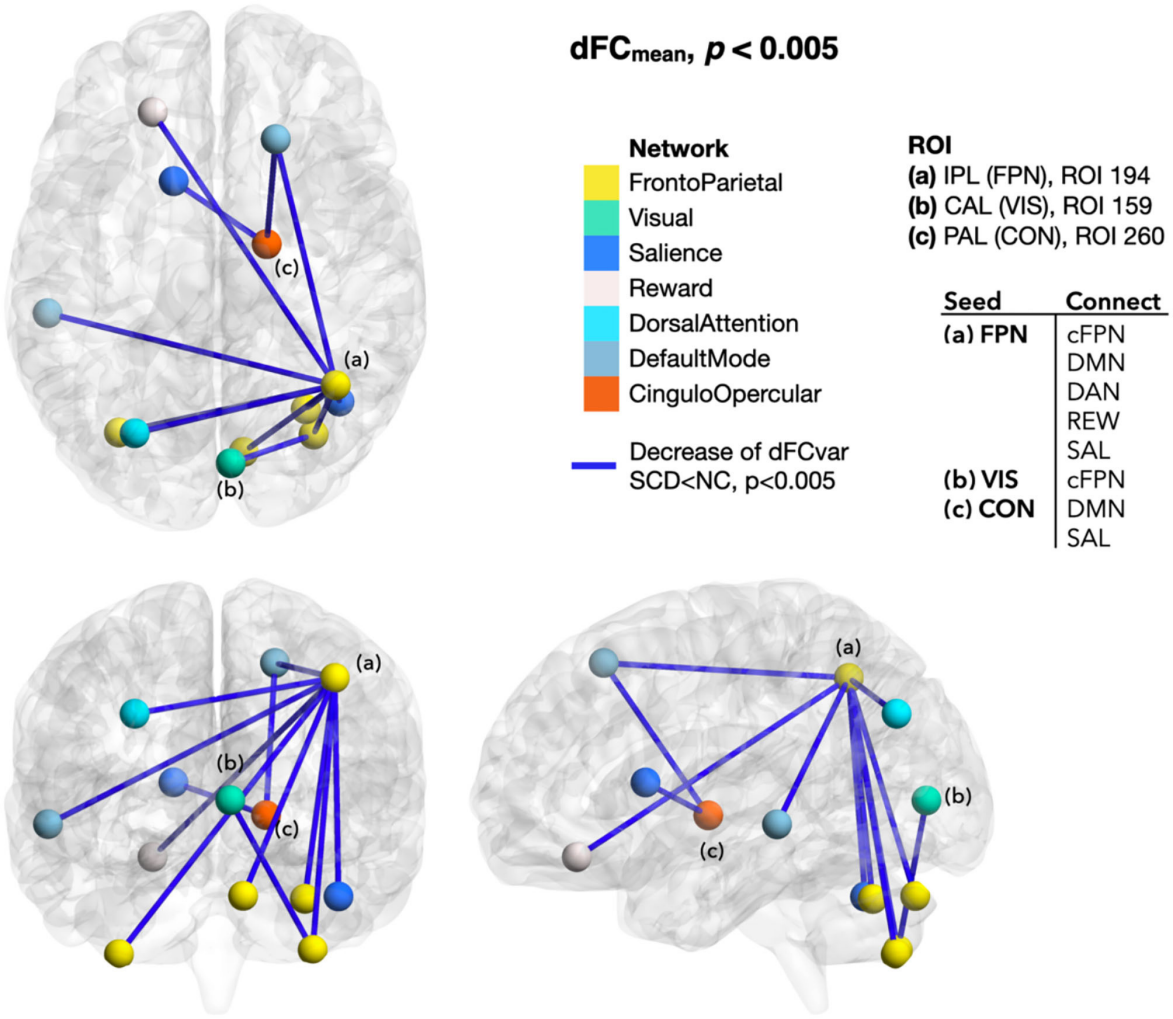
Differences of dFC_mean_ between SCD and NC. The dFC_mean_ seeded from the ROIs with local mdALFF group differences generally decreased in SCD than NC, including the dFC from the FPN ROI to the cFPN, DMN, DAN, REW, and SAL ROIs (*p* < 0.005). Color of line indicated the changes of dFC. Blue was for decrease. Color of ROIs represented the functional network they belonged to. dFC_mean_, mean of the dynamic functional connectivity; SCD, subjective cognitive decline; NC, normal control; ROI, region of interest; IPL, inferior parietal lobule; FPN, frontoparietal network; CAL, calcarine area; VIS, visual network; PAL, pallidum of lenticular nucleus; CON, cingulo-opercular network; cFPN, frontoparietal network in the cerebellar regions; DMN, default mode network; DAN, dorsal attention network; REW, reward network; SAL, salience network.

**Figure 5:**
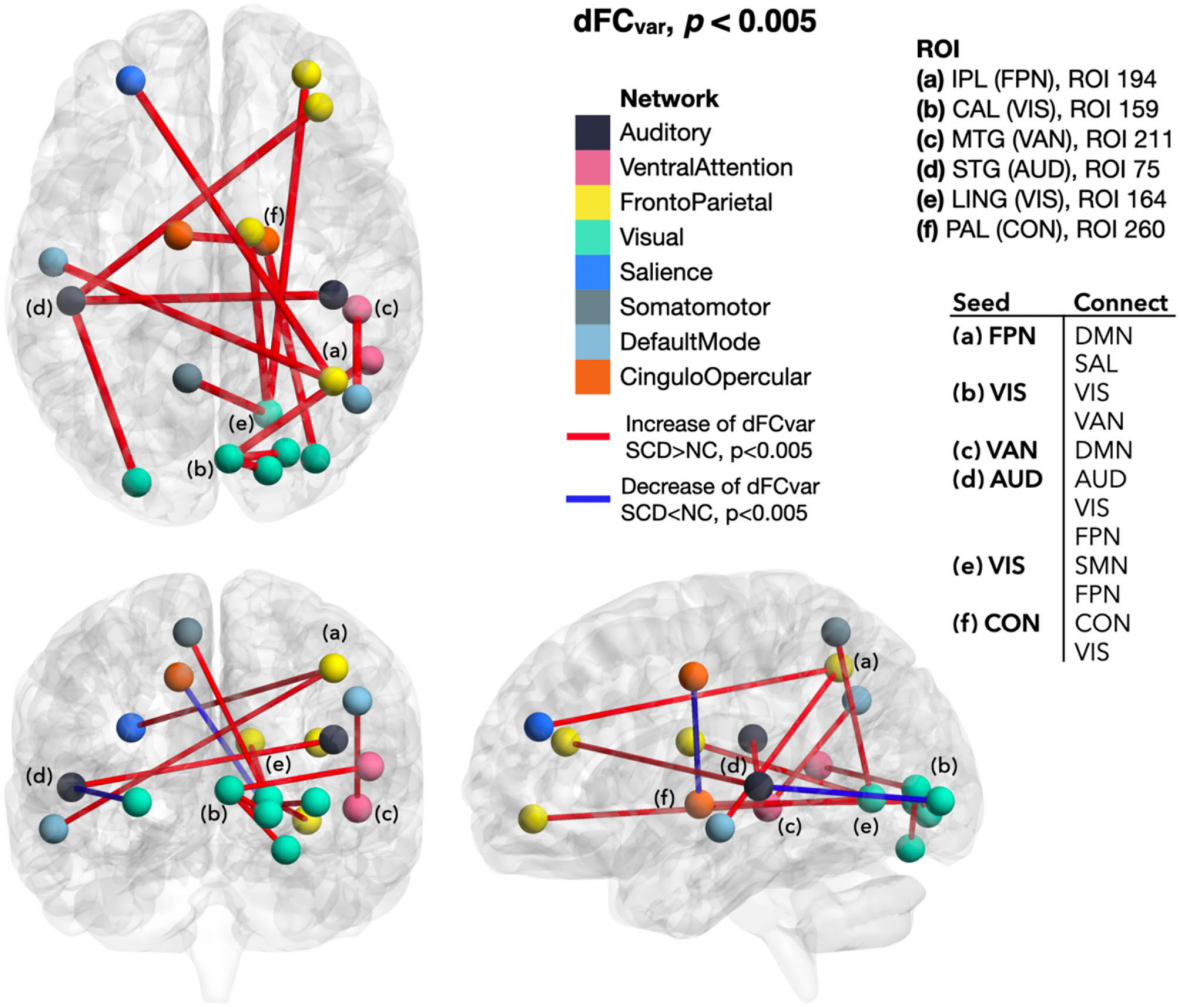
Differences of dFC_var_ between SCD and NC. In SCD, dFC_var_ generally increased from the ROIs with local dynamic connectivity changes (seeding ROIs) to other brain regions. In contrast, the dFC_var_ decrease appeared between the ROIs of AUD-VIS and within CON. dFC_var_, variance of the dynamic functional connectivity; SCD, subjective cognitive decline; NC, normal control; ROI, region of interest; IPL, inferior parietal lobule; FPN, frontoparietal network; CAL, calcarine area; VIS, visual network; MTG, middle temporal gyrus; VAN, ventral attention network; STG, superior temporal gyrus; AUD, auditory network; LING, lingual gyrus; VIS, visual network; PAL, pallidum of lenticular nucleus; CON, cingulo-opercular network; DMN, default mode network; SAL, salience network; SMN, somatomotor network.

In SCD than NC, the extended dFC from those regions with altered local dynamic connectivity generally showed a lower mean and higher variance of functional connectivity dynamics that indicated weakened and unstable brain activity. Although the between-group NBS did not reach a significant difference, the dFC values between SCD and NC groups showed tendencies of early functional connectivity changes in SCD. To be specific, dFC_mean_ decreased between the ROIs of FPN-cFPN, FPN-the default mode network (DMN), FPN-the salience network (SAL), FPN-the dorsal attention network (DAN), FPN-the reward network (REW), VIS-cFPN, CON-DMN, and CON-SAL (*p* < 0.005; Figure 4). In addition, increased dFC_var_ was found between the ROIs of FPN-DMN, FPN-SAL, VAN-DMN, VIS-VAN, VIS-the somatomotor network (SMN), VIS-FPN, AUD-VIS, AUD-FPN, CON-VIS, within CON, within VIS, and within AUD (*p* < 0.005; Figure 5).

### Correlations of mdALFF to Clinical Indices

In correlation analysis, AD8 score for the degree of SCCs was negatively correlated with the mdALFF_mean_ in right IPL of FPN (*r* = −0.29), but positively correlated with the mdALFF_mean_ in right MTG of VAN (*r* = 0.30), and right CAL of VIS (*r* = 0.38). AD8 score was also positively correlated with the mdALFF_var_ in left STG of AUD (*r* = 0.51), right LING of VIS (*r* = 0.42), and right MTG of VAN (*r* = 0.32). Statistical significance was set at *p* < 0.05 after FDR correction (Table 2).

**Table 2:** Partial correlation coefficient (*r*) of mdALFF_mean_ and mdALFF_var_ to clinical indices.

**Location^†^**	**mdALFF**_**mean**_			**mdALFF**_**var**_			
	Figure 2			Figure 3			
	**ROI(a)**	**ROI(b)**	**ROI(c)**	**ROI(a)**	**ROI(b)**	**ROI(c)**	**ROI(d)**
Hemisphere	Right	Right	Right	Left	Right	Right	Right
AAL location	IPL	MTG	CAL	STG	LING	MTG	PAL
Network	FPN	VAN	VIS	AUD	VIS	VAN	CON
ROI number	194	211	159	75	164	211	260
AD8	−0.29^*^	0.30^*^	0.38^*^	0.51^*^	0.42^*^	0.32^*^	0.19
MMSE	0.29^*^	0.01	−0.19	−0.20	0.10	−0.07	−0.13
MoCA	0.14	−0.14	−0.08	−0.21	−0.04	−0.07	−0.19
DSC	0.01	−0.01	0.04	0.02	−0.05	−0.02	−0.25
DST-forward	0.14	−0.04	−0.02	−0.21	−0.10	−0.07	0.01
DST-backward	0.10	−0.03	−0.08	−0.06	0.02	−0.11	0.02
LNS	−0.01	−0.09	0.10	−0.05	0.03	−0.18	−0.17
CF-animal	0.13	0.15	−0.01	−0.08	−0.23	−0.06	−0.18
CF-fruit	0.32^*^	0.11	−0.09	−0.10	−0.15	−0.12	−0.07
CF-color	0.13	−0.07	0.00	−0.09	−0.11	−0.25	−0.06
CF-city	−0.19	−0.04	0.12	0.08	−0.14	−0.06	−0.21
FMT	0.20	−0.05	−0.07	−0.04	0.02	−0.07	−0.11
HADS-A	−0.20	0.17	0.10	0.14	0.21	0.33^*^	0.11
HADS-D	0.17	−0.10	−0.02	0.22	0.12	0.23	0.13

* *Partial correlation coefficient with a FDR-corrected p < 0.05. The statistically significant values were in bold type.*

† *References to ROI location in Figures 2, 3. Between-group differences of partial correlation coefficient were tested by Fisher's Z-transformation and one-tailed test. Boxes marked the correlations with significant group differences between SCD and NC (FDR-corrected p < 0.05).*

*The following partial correlation coefficients of mdALFF_var_ to cognitive scores differed between the SCD and NC groups: mdALFF_var_ in right MTG of VAN with MMSE score (SCD r = 0.398 vs. NC r = −0.108), mdALFF_var_ in right PAL of CON with CF-fruit score (r = 0.297 vs. −0.275) and CF-city score (r = −0.293 vs. r = 0.325). In addition, the correlations of the HADS-D depression score were different in the following local dynamic connectivity: mdALFF_mean_ in right IPL of FPN (r = 0.197 vs. −0.271), mdALFF_mean_ in right CAL of VIS (r = −0.237 vs. 0.187), mdALFF_var_ in left STG of AUD (r = −0.294 vs. 0.235)*.

*ROI, region of interest; AAL, automated anatomical labeling; MMSE, Mini-Mental State Examination; MoCA, Montreal Cognitive Assessment; DSC, digit symbol coding; DST, digit span test; LNS, letter-number sequencing; CF, category fluency; FMT, facial memory test; HADS-A, Hospital Anxiety and Depression Scale—Anxiety subscale; HADS-D, Hospital Anxiety and Depression Scale—Depression subscale; mdALFF, mean dynamic amplitude of low-frequency fluctuation; var, variance; IPL, inferior parietal lobule; MTG, middle temporal gyrus; CAL, calcarine fissure and surrounding cortex; STG, superior temporal gyrus; LING, lingual gyrus; PAL, pallidum of lenticular nucleus; FPN, frontoparietal network; VAN, ventral attention network; VIS, visual network; AUD, auditory network; CON, cingulo-opercular network; FDR, false discovery rate; SCD, subjective cognitive decline; NC, normal control*.

For the correlations with cognitive performance, mdALFF_mean_ in right IPL of FPN was correlated positively with MMSE score (*r* = 0.29, FDR-corrected *p* < 0.05) and CF score of fruit category (*r* = 0.32, FDR-corrected *p* < 0.05) (Table 2).

In addition, mdALFF_var_ in right MTG of VAN was associated with the degree of anxiety, in terms of HADS-A (*r* = 0.33, FDR-corrected *p* < 0.05) (Table 2).

For the group differences of mdALFF-clinical correlation, different trends of correlations between the SCD and NC groups were observed between MMSE score to mdALFF_var_ in right MTG of VAN, CF scores for fruit and city categories to mdALFF_var_ in right PAL of CON, HADS-D scores to mdALFF_mean_ in right IPL of FPN, mdALFF_mean_ in right CAL of VIS, and mdALFF_var_ in left STG of AUD (FDR-corrected *p* < 0.05) (Table 2).

## Discussion

### Summary

This was an rs-fMRI study of local mdALFF and related extension of dFC in community-dwelling cognitively normal older adults, who were divided into the SCD and NC groups for comparisons. In SCD, ROI-based mdALFF local dynamic connectivity analysis showed mixed changes in mean and variance. The ROIs with significant group differences of mdALFF_mean_ and mdALFF_var_ were mainly located in the right cerebral hemisphere. In addition, the alterations of intrinsic brain activity of SCD were toward a regional-specific mixture of deficiency-compensation mechanisms and unstable local dynamics. In detail, local dynamic connectivity became weak in FPN for central executive control and cognitive flexibility. Still, FPN balanced both subjective and objective cognition and had a crucial role in cognitive reserve in preclinical dementia. In contrast, functional compensation of SCD started in visual and attention networks. To sum up, the temporal fluctuations of local BOLD signals explained regional brain changes of SCD, which responded to the subtle cognitive decline and tried to resume the homeostasis of the brain.

The dFC from those ROIs with local dynamic changes also differed between SCD and NC. Those regions with altered mdALFF_mean_ of VIS, FPN, and VAN showed decreased dFC_mean_ and increased dFC_var_ to other brain regions. An increased variance indicated instability and fluctuation of local dynamic connectivity. The CON, VAN, and AUD regions with increased mdALFF_var_ also showed unstable outward connections with an increased dFC_var_. The altered dFC in SCD could result from disconnection and noise signals due to early degeneration or resource relocation to cope with the subtle cognitive decline.

### The Cognitive Theories of Aging Help Interpret the Altered Dynamic Connectivity in SCD

The brain's responses to the emerging neurodegeneration and functional deterioration of SCD resembled accelerated aging (Dennis and Thompson, 2014; Chen and Arai, 2020). For example, homeostatic disinhibition by decreased GABAergic transmission in response to the defected and inefficient glutamatergic synaptic transmission is a common pathway of aging and AD (Gleichmann et al., 2011). In addition, functional compensation is one of the central theories for explaining the continuous brain changes of aging (Grady, 2012) and preclinical dementia (Jessen et al., 2014; Viviano and Damoiseaux, 2020). Overactivation of bilateral prefrontal cortex in working memory tests is considered compensations to aging in the model of *hemispheric asymmetry reduction in older adults (HAROLD)* (Cabeza, 2002). However, compensations could not fully cover the complex brain changes. Other explanations for the regionally increased brain activity include the *dedifferentiation* that reduced resource allocation and inefficient neural responses result in non-selective brain activation (Li and Lindenberger, 1999) and the *scaffolding theory of aging and cognition (STAC)* that additional networks are recruited in response to the functional decay of the initial network (Park and Reuter-Lorenz, 2009).

In recapitulating the above ideas, a highly differentiated brain reacts dynamically to the emerging and progressive destruction due to neurodegeneration. A comprehensive evaluation of behavioral, information-processing, and neurobiological evidence and referencing it to the cognitive theories of aging helps us to understand the mechanisms of transition from normal to cognitively impaired states (Li et al., 2001).

### History of Functional Connectivity Dynamics Study and What Did This Study Add to Current Knowledge?

Blood oxygenation level-dependent signals and resting-state networks have initially been considered in static states but later discovered to be fluctuating overtimes (Chang and Glover, 2010). It opened a new horizon of using BOLD dynamics to characterize disease-related brain changes in different scales from local changes, network reorganization to global brain remodeling (Biswal, 2012). By sliding-window technique, dynamic analysis of rs-fMRI can simulate neural activities of the brain by a comparable temporal resolution as that of electroencephalogram (EEG). Simultaneous resting-state EEG-fMRI showed distinct correlations between EEG power spectra and dynamic fluctuations of resting-state networks (Laufs, 2008; Hutchison et al., 2013). In addition, simultaneously recording of local field potential and fMRI found the dependence of functional connectivity dynamics to the behavior states (Hutchison et al., 2013; Pan et al., 2013). Lately, the development of analytic methods further extended the applications of sliding-window techniques to pairwise dFC comparisons, dynamic graph analysis, frame-wise analysis, state modeling, and temporal modeling. After that, dynamics of functional connectivity becomes a vital feature other than static connectivity for describing how the brain works (Preti et al., 2017).

In 2012, Jones et al. proposed the non-stationary modular architecture model of the resting brain and demonstrated temporal differences of DMN sub-network configurations in patients with AD and age-matched healthy controls. According to it, the resting brain in AD pronged to stay in the state emphasizing prefrontal anterior DMN but spend less time in the state weighting on posterior DMN (Jones et al., 2012). After that, several studies successively showed the dynamic features of AD (Gu et al., 2020), MCI (Wee et al., 2016; Jie et al., 2018), SCD (Xie et al., 2019; Dong et al., 2020; Yang et al., 2020; Chen et al., 2021), and across the AD spectrum (Cordova-Palomera et al., 2017; Demirtas et al., 2017).

The dynamics of functional connectivity are continuously changing over disease progression in the AD continuum. Combining temporal and spatial variability, dynamic features of resting-state networks can distinguish early MCI from late MCI and early MCI from healthy controls (Jie et al., 2018). It found the earliest changes in functional connectivity dynamics and its evolution over the trajectories of cognitive decline. Indeed, later studies of SCD confirmed that altered functional connectivity dynamics began in the preclinical phase. A large-scale brain dynamic study based on the graph theory described the temporospatial dynamics of SCD. Temporal flexibility and spatiotemporal diversity characterized SCD differently from NC and outperformed sFC and structural metrics in the support vector machine (SVM) classifier. This study also showed a mixed increase and decrease of connectivity dynamics across brain regions in SCD (Dong et al., 2020). Another state-modeling study of SCD found more dwell time in a state of hypoconnectivity within and between networks but less time in hyperconnectivity within and between the auditory, visual, and SMN. These altered dFC properties are further correlated significantly with cognitive performance (Chen et al., 2021). The other graphic analysis also revealed altered correlations between centrality frequency and cognitive performance in SCD when compared to NC. The correlation decreased in the anterior brain but increased in the posterior brain, especially significant within DMN (Xie et al., 2019).

The functional connectivity dynamics also change at the local level. A dynamic ALFF (dALFF) analysis comparing people with subjective memory complaints, NC, and MCI found lower regional dynamic connectivity in the hippocampus, parahippocampal gyrus, fusiform gyrus, precuneus, paracentral gyrus, and cerebellum in SCD. Furthermore, general concordance was higher in those with subjective memory decline than in NC (Yang et al., 2020). That was, the local dynamics of vulnerable areas became less active in SCD, and general connectivity dynamics became more synchronized and less favoring normal arousal state (Yan et al., 2017). Compared to this dALFF study of subjective memory decline (Yang et al., 2020), enrolling criteria of our study included multi-domain self-awareness of impaired cognitive abilities more than solely memory concerns. Our comparisons of mdALFF between SCD and NC identified more brain regions of various functional networks than the isolated memory-related regions as in the dALFF study. In addition, our analyses focused on network properties of the ROIs toward an explainable result with clinical relevance.

In summary, previous studies and our study of connectivity dynamics in SCD show the changes in both temporal and spatial aspects and from global to local scales. The dynamic disconnection moves the resting brain of SCD into a less coordinated state. With the correlations of clinical indices, regional connectivity dynamics are transformed into neuroimaging markers for indicating the transitional characteristics of the brain from a normal healthy state to the initiation of the AD spectrum disorders.

### Right Brain Changes More Than Left Brain

Interestingly, the ROIs with mdALFF changes in SCD were primarily located in the right cerebral hemisphere. Even the marginal changes occurred more on the contralateral left cerebellar hemisphere that connects through cerebro-cerebellar circuits to the right cerebrum. We presume this result echoes the rightward functional lateralization phenomenon in aging and AD neurodegeneration.

As early as 2002, Cabeza established the *HAROLD* model for the aging brain that describes asymmetric functional reduction with a more significant functional loss on the left than the right cerebral hemisphere. As it states, the brain activation is more bilateral in older people than left-lateralized in younger adults regarding perception, inhibitory control, and memory function (Cabeza, 2002). Previous sFC studies of AD spectrum disorders also documented the similar right lateralization phenomenon of functional connectivity as in the aging brain. Functional deviation to the non-dominant hemisphere in MCI and AD suggests primary dysfunction of the dominant left hemisphere and corresponding compensatory functional shift to the right hemisphere (Liu et al., 2018). Furthermore, the regional cerebral metabolic rate of glucose on FDG-PET decreased more in the left hemisphere than the right hemisphere in patients with MCI and AD than in normal people (Weise et al., 2018). The asymmetric reduction in glucose metabolism in the left hemisphere may be the underlying physiological reason for the function deviation to the right hemisphere.

In this study, we focused on the SCD stage, the transition between normal and MCI. The dynamics of functional connectivity showed that the local disconnection and compensatory gain of function emerged in the non-dominant right hemisphere. Before influencing the dominant hemisphere, preclinical neurodegeneration affects first on the non-dominant hemisphere. Thus, the non-dominant cerebral hemisphere and its related contralateral cerebellar hemisphere are the precursors of functional connectivity changes. To sum up the findings of previous AD and MCI studies and our SCD study, the non-dominant right hemisphere shows early preclinical dynamic connectivity changes in the SCD stage. When cognitive decline progresses to the MCI and AD stages, the dominant left hemisphere loses function, and the non-dominant right hemisphere takes the response to rescue the functional loss.

### Properties of Networks May Affect Their Vulnerability to Early Neurodegenerative Processes

We found that FPN (i.e., the central executive network, CEN) lost local dynamic connectivity in SCD. Other studies demonstrated that dynamic connectivity of DMN is affected in SCD (Xie et al., 2019). From the network property study, the properties of each network are specific for its unique functions. For example, DMN and FPN/CEN are the internally driven functional networks for higher-order mental processes. Their high integration and low coherence are in terms of high between-network connectivity, low within-network connectivity, low dynamic metastability, and low dynamic synchrony (Lee and Frangou, 2017). Furthermore, activation of FPN and DMN in task fMRI is associated with the subtle cognitive decline in cognitively normal non-MCI elderly (Zanchi et al., 2017). In our study, the regions of FPN, cFPN, and cDMN showed significant or marginal mdALFF_mean_ decreases in SCD than NC. The within-network dFC_mean_ of cerebral-cerebellar parts of FPN (FPN-cFPN) also decreased in SCD. We proposed that the above networks are complicated and delicate in connecting outward and may be more vulnerable to disconnection.

In contrast, the other networks have low between-network connectivity, such as VIS and SMN (Lee and Frangou, 2017). They may contribute to functional compensation in the early stages of the AD continuum because they are relatively functioning well and can rescue the functional loss of the rest of the brain. These primary networks can work on their own and rely less on inter-network functional linkage. As in our study, mdALFF_mean_ of CAL of the primary visual cortex increased while its dFC to the cFPN region decreased. Another dynamic rs-fMRI study of SCD also showed reduced dwell time of hyperconnectivity between the primary networks (i.e., VIS, AUD, and SMN) than controls (Chen et al., 2021). Therefore, we reasoned that the primary networks work more isolated in SCD. The decentralized discrete operation is the alteration in response to the early neurodegeneration.

### Functional Networks Altered in SCD

#### FPN and CFPN That in Charge of Central Executive Control

Fluid intelligence, including cognitive processing speed, working memory and long-term memory, is in contrast to crystalized intelligence regarding world knowledge and vocabulary (Park and Bischof, 2013). In normal aging, fluid intelligence declines with age, whereas crystallized intelligence remains at the same level. Similarly, fluid intelligence decreases more rapidly in preclinical AD than crystallized intelligence. The gap of decline between the two categories of intelligence is associated with amyloid deposition in the brain (Mcdonough et al., 2016).

Frontoparietal network is a flexible hub for flexible brain coordination for cognitive control (Zanto and Gazzaley, 2013) and adaptive task control (Cole et al., 2013). In addition, FPN maintains fluid intelligence by functional integration to the rest of the brain (Cole et al., 2015). However, FPN is affected early in preclinical AD. An rs-fMRI study of SCD demonstrated positive correlations between the comprehensive cognitive performance by the MoCA score and the static local connectivity of FPN (Wang et al., 2019). Well-functioning executive control is crucial to maintain cognitive preservation in the preclinical stage of dementia before the development of cognitive impairment.

In our study, the regions of FPN showed decreased mdALFF_mean_ in SCD. The regions of cFPN also showed marginally decreased mdALFF_mean_ and mdALFF_var_ in SCD. These regions of FPN and cFPN have located in the cerebral association cortices and its mirroring posterior cerebellum, which are connected by cerebro-cerebellar circuits for reciprocal control of higher-order cognitive function (Buckner et al., 2011). Furthermore, dFC_mean_ showed decreased changes between the regions of FPN and cFPN in SCD than NC. The altered local and long-range dynamic connectivity in SCD represents the vulnerability of the higher-order functional networks and the related reduction in central executive control and cerebellar forward controlling. In correlation analysis, the regional mdALFF_mean_ within FPN is correlated negatively with the degree of subjective cognitive concerns by the AD8 score and positively with cognitive performance in terms of the scores of MMSE and CF of fruit. Therefore, the local dynamics of FPN are crucial in balancing subjective and objective cognition in preclinical dementia, even if the cerebral-cerebellar disconnection within the central executive controls system has begun.

#### The Triple Network Dysfunction in SCD: FPN, DMN, and SAL/CON

In the triple network model for cognitive and affective dysfunction, SAL weights the external stimuli or internal awareness to balance the switches between cognitive controlling FPN/CEN and the self-referential DMN (Menon, 2011). The anterior cingulate cortex and anterior insula are the main parts of SAL to detect salient stimuli, coordinate, and dynamically allocate neural resources (Uddin, 2015). Besides, CON and SAL are considered equivalent or adjacent networks that CON has closer collaborations with FPN (Gratton et al., 2018).

With this background knowledge, we revisit the mdALFF and dFC results. The decreased local mdALFF within FPN, cFPN, and cDMN and the weakened outward dFC between FPN-DMN, FPN-SAL, CON-SAL, and CON-DMN suggest an imbalanced triple network organization centering the decline of FPN local connectivity in SCD. In addition, the regional FPN mdALFF_mean_ is correlated with subjective and objective cognition. Similarly, in a study of dFC based on the triple network model, Xue et al. revealed aberrant dFC variability among DMN, SAL, and executive control network (i.e., FPN) in SCD and MCI compared to NC; moreover, the dFC variability within FPN is correlated with cognitive performance (Xue et al., 2021). To recap our study and Xue's study, the triple network model's aberrant dynamic activity and disorganization explain the subtle cognitive decline and awareness of cognitive impairment in SCD. These delicate dynamic changes in the triple networks appear early than the overt rise of cross-network functional connectivity in late MCI and AD (Li et al., 2019).

#### Attention Networks in AD Spectrum

The VAN detects unexpected stimuli and triggers attention shifting (bottom-up stimulus-driven attention), in cooperating with the goal-relevant top-down attention by the dorsal attention network (Corbetta and Shulman, 2002; Fox et al., 2006). Earlier in the AD continuum, VAN is preserved but DAN is affected in the MCI stage. However, both DAN and VAN are impaired in more advanced stages, as in the dementia stage (Sorg et al., 2007; Qian et al., 2015; Wang et al., 2019). As our results also suggested, the bottom-up attention maintained by the VAN was intact in the SCD stage. It even showed compensatory efforts for preserving cognitive performance in the SCD stage earlier before the development of MCI. In summary, when the top-down attention processing is impaired in the preclinical and MCI phases of dementia, the bottom-up attention processing retains or raises to keep the performance level.

#### Visual Network

The visual network controls visual signal processing, visual memory, visual learning, and visual coordination. A compensatory increase of sFC in SCD has been noticed in the medial visual network (Hafkemeijer et al., 2013) and inferior and medial occipital regions (Sun et al., 2016). In our study, the increased local dynamic connectivity was also located in the medial visual network, with increased mdALFF_mean_ and mdALFF_var_ of calcarine and lingual areas. The increase of visual local dynamic connectivity suggests a visual compensation for the subtle cognitive decline of SCD. In addition, the degree of SCCs was associated with this local dynamic connectivity. In addition, another rs-fMRI study also showed associations of subjective memory complaints and the sFC of the visual cortices, including the cuneus and lingual gyrus (Kawagoe et al., 2019). Therefore, the visual compensatory mechanism is one of the early modifications in response to subtle cognitive decline.

#### Auditory Network

The auditory cortex is one of the primary perception areas of the brain. The central auditory system receives external signals from peripheral auditory structures, and the auditory functional network operates the hearing-related cognitive processes. Auditory cognition includes auditory working memory, auditory semantic knowledge, auditory object recognition, auditory spatial processing, and auditory scene analysis (Johnson et al., 2021). Moreover, the auditory functional network does not work alone, but interplays with frontoparietal executive control and sensory networks for cognitive processes of auditory working memory (Kaiser, 2015).

In AD spectrum disorders, central auditory system dysfunction leads to distortion of the hierarchical auditory processing, loss of auditory plasticity, and impairment of auditory reciprocity. It results in a series of auditory cognitive deficits (Johnson et al., 2021). In a pathological inspection of the brains of patients with AD, senile plaques and neurofibrillary tangles were distributed throughout auditory-related nuclei (Sinha et al., 1993). In patients with AD, deficits of the auditory spatial processing are associated with gray matter volume loss, especially the posterior cortical atrophy (Golden et al., 2015). From functional aspects, the decrease of intra-network resting-state functional connectivity of the auditory network advances from normal controls, early MCI, to late MCI (Zhang et al., 2018).

In our study, the local dynamic connectivity mdALFF_mean_ decreased marginally at the right ROL (*p* = 0.006) and the mdALFF_var_ increased at the left STG in SCD (*p* < 0.001). In previous studies, right ROL was one of the brain regions that showed early functional changes in AD spectrum disorders. For example, the right ROL local property and its subgraph connecting to the right insula could differentiate early MCI from NC (Cui et al., 2018). Furthermore, our study showed that the dFC_var_ within AUD-AUD and between AUD-FPN regions increased significantly in SCD than NC. The unstable intra- and inter-network collaborations of the central auditory system signify the preclinical changes in SCD and correspond to the cognitive maintenance co-played by auditory and executive networks (Kaiser, 2015).

### Limitations

There are several limitations of this study. First, previous studies have shown heterogeneous results of functional connectivity changes in SCD, which may be related to obtaining study groups at different phases of SCD (Viviano and Damoiseaux, 2020; Wang et al., 2020). The hypothetic model of functional connectivity changes in SCD includes a first increase of functional connectivity due to noisy signal propagation and compensation, followed by a later decrease due to the progression of disconnection (Viviano and Damoiseaux, 2020). The functional connectivity changes are continuous processes even in the SCD stage. Different research groups can enroll participants at either early SCD, late SCD, or a heterogeneous population and yield inconsistent findings. In our case, longitudinal follow-up will be a chance to know the sequential changes in functional connectivity dynamics of this community-dwelling group of people with SCD. Second, this study enrolled only SCD and NC. No advanced stages of the AD spectrum were included for comparisons. The setting of community-based research limited our studying population. Future enrollments of MCI and dementia groups from hospital-based settings will overcome this limitation and increase the generalizability of our findings to the continuum of AD. Finally, the sample size was relatively small when aiming to discover subtle brain changes in preclinical dementia, and therefore, the testing for multiple comparisons did not provide strong evidence. Thus, increasing sample size in future works is warranted to bring more convincing results for the dynamic connectivity changes in SCD.

## Conclusions

Dynamic features of functional connectivity evolve along the trajectories of cognitive decline. This rs-fMRI study shows that the dynamic changes start early in the preclinical SCD stage. Local dynamic connectivity decreased in the regions of FPN but increased in VIS and VAN. Mixed weakening, compensatory enhancing, and unstable local dynamic connectivity mdALFF and their outward dFC suggest the transitional nature of the SCD stage from normal to cognitive impairment. Thus, the brain keeps a dynamic balancing between functional maintenance and subtle cognitive impairment in this stage. The altered local dynamics were associated with the degree of subjective cognitive concerns. In addition, the local dynamics of the right IPL of FPN are correlated with subjective and objective cognition and may be crucial for cognitive preservation in preclinical dementia.

## Data Availability Statement

The raw data supporting the conclusions of this article will be made available by the authors, without undue reservation.

## Ethics Statement

The studies involving human participants were reviewed and approved by the Institutional Review Board of Chang Gung Memorial Hospital. The patients/participants provided their written informed consent to participate in this study.

## Author Contributions

Y-CW: conceptualization, methodology, data curation, formal analysis, investigation, resource, writing—original drafting, visualization, project administration, and funding acquisition. Y-CK: methodology, software, formal analysis, writing—original drafting, and visualization. W-YH: resource, data curation, and funding acquisition. CL: data curation, project administration, and funding acquisition. Y-LC: data curation and supervision. C-KC: resource, supervision, and project administration. Y-CS: data curation and resource. C-PL: conceptualization, methodology, investigation, resource, writing, review, editing, and supervision. All authors contributed to the article and approved the submitted version.

## Funding

This research was supported by the grants of the Chang Gung Research Project to Y-CW (CRRPG2G0072), W-YH (CRRPG2K0032), C-PL (CRRPG2G0062 and CRRPG2K0022), C-KC (CRRPG2G0052 and CRRPG2K0012), and the Community Medicine Research Center of Keelung Chang Gung Memorial Hospital (CLRPG2J0011 and CLRPG2L0052). This research was also supported by grants to C-PL from the Ministry of Science and Technology (MOST) of Taiwan (MOST 110-2321-B-010-004, MOST 110-2634-F-010-001, MOST 111-2321-B-A49-003, and MOST 108-2321-B-010-010-MY2).

## Conflict of Interest

The authors declare that the research was conducted in the absence of any commercial or financial relationships that could be construed as a potential conflict of interest.

## Publisher's Note

All claims expressed in this article are solely those of the authors and do not necessarily represent those of their affiliated organizations, or those of the publisher, the editors and the reviewers. Any product that may be evaluated in this article, or claim that may be made by its manufacturer, is not guaranteed or endorsed by the publisher.
